# Achievements and Challenges of Classical Swine Fever Eradication in Brazil

**DOI:** 10.3390/v12111327

**Published:** 2020-11-19

**Authors:** Luís Guilherme de Oliveira, Igor Renan Honorato Gatto, Marina Lopes Mechler-Dreibi, Henrique M. S. Almeida, Karina Sonálio, Gabriel Yuri Storino

**Affiliations:** 1 School of Agricultural and Veterinarian Sciences, São Paulo State University (Unesp), Jaboticabal, Via de Acesso Prof. Paulo Donato Castelanne s/n, Jaboticabal 14884-900, SP, Brazil; mlopesvet@gmail.com (M.L.M.-D.); h.almeida@unesp.br (H.M.S.A.); karina.sonalio@unesp.br (K.S.); gabrielystorino@gmail.com (G.Y.S.); 2 Ourofino Animal Health Ltda. Rodovia Anhanguera SP 330, Km 298, Distrito Industrial, Cravinhos, São Paulo 14140-000, Brazil; igatto_10@hotmail.com

**Keywords:** classical swine fever infection, outbreaks, domestic pigs, wild boars, South America

## Abstract

Classical swine fever virus (CSFV) causes one of the most critical diseases in the porcine industry worldwide. In Brazil, the first description of the infection was reported in 1888, and the national recognition of the first free zone (FZ) occurred in 2001. Brazil has been recently recognized (2015–2016) by the World Organisation for Animal Health (OIE) with an FZ involving 15 states and the Federal District, corresponding to 95% of the industrial production of pigs in the country, and a non-free zone (NFZ), comprised by the North and Northeast regions of the country, with approximately 18% of the national pig herd and 5% of industrial production. This review aims to describe the history, the control and eradication actions, the recent occurrence of outbreaks in the NFZ, and the results obtained by the surveillance systems’ action in the FZ for CSF in Brazil since its creation. In the passive surveillance system, the notification of the suspect cases of classical swine fever (CSF) is mandatory while in the active surveillance system adopted in the FZ consists of serological monitoring of certified swine breeding farms (CSBFs), intensive pig farming (IPF), non-technified pig herds (NTPig), surveillance in slaughterhouses and monitoring the populations of wild pigs. In this region, the last outbreaks of the disease occurred in 1998, while in the NFZ, 28 outbreaks were detected from 2005 to 2017, with an apparent lethality rate of 93.96% (840/894). However, in 2018 and 2019, 68 new outbreaks were registered with an apparent lethality rate of 75.05% (1095/1459). Therefore, in 2019, the Brazil CSF-Free Strategic Plan was created to eradicate the infection from the country’s NFZ, since outbreaks in this region present a risk of reintroducing the disease FZ. Finally, differences in characteristics between the regions show factors that still need to be considered for the construction of a robust surveillance system in the NFZ and some improvements in the FZ. Thus, the control of CSF throughout the Brazilian territory requires strict sanitary guidelines, promoting animal health and, consequently, the national production chain’s competitiveness.

## 1. Introduction

Classical swine fever (CSF) is one of the most important infectious viral disease of domestic pigs and wild boars, caused by the classical swine fever virus (CSFV), which belongs to the species *Pestivirus* C, is an enveloped RNA virus belonging to the growing genus *Pestivirus*, within the family *Flaviviridae* [1,2,3]. CSFV strains can be divided into three genotypes, with three to four sub-genotypes [4]. In the American continent, the circulating viruses belong to genotype 1, with only the 1.1 strains reported in Argentina, Brazil, Colombia, and Mexico [5]. Even though only genotype 1 is reported in Brazil, we should consider that viral replication depends on RNA polymerases, which lack proofreading. Therefore, the occurrence of mutations and the emergence of highly virulent CSFV could occur [6].

CSF has a considerable impact on animal health and the swine industry; therefore, it is a mandatory reporting disease for the World Organisation for Animal Health (OIE) [1]. The infection was first recognized in the early 19th century, and its viral etiology was only established in the early 20th century [7]. The virus has spread throughout the world, causing severe pig farming losses with several consequences for the countries’ economies. Besides, CSF still occurs in many countries in South and Central America, parts of Eastern Europe and neighboring countries, and Asia, including India [4]. However, little is known about the infection situation in the African continent. Furthermore, after the implementation of strict control measures, the infection has been eradicated in many territories, such as the United States, Canada, New Zealand, and Australia [8].

Control and prevention are based on reliable diagnosis, elimination of the infected animals, the establishment of restriction zones, movement restrictions, and tracking of possible contacting animals [9]. Prophylactic vaccination and other treatments are questionable and sometimes even prohibited. That is why vaccination against CSFV depends on the disease’s epidemiology, the animals affected, and the economic situation, so it should be cautiously indicated [6]. In some countries with significant pig production CSF remains or is sporadically present, which may be associated with the limited implementation of prophylaxis actions [7,10], despite maintaining a government structure for surveillance and control. Another challenge concerns the pigs and wild boar populations, as they are an important reservoir for the virus and source of infection for reintroduction in the domestic swine herds [11].

Currently, Brazil is the fourth largest pork producer and exporter in the world. In 2018, with a herd of approximately two million sows and 40 million pigs for slaughter, it was responsible for 3% of the production and 10% of the world exports to more than 70 countries and moving approximately R$150 billion [12]. Still, the pig production in Brazil has been growing, with an essential international competitiveness. However, the maintenance and opening of Brazilian pork markets is fundamental to the activity’s economic viability and depends, beyond quality standards, on a certified and internationally recognized health condition [13]. In this scenario, CSF outbreaks in the free zone would affect one of the most important economic activities in Brazilian agribusiness.

Currently, Brazil has a CSF free zone (FZ) that comprises approximately 95% of all industrial pig production. On the other hand, there is the occurrence of CSF outbreaks limited to a few States of the North and Northeast regions, classified as CSF non-free zone (NFZ). Regarding territorial extension, the NFZ is expressive and threatens the country’s position in the international market, and the infection compromises local communities with subsistence production [14]. If infection occurs in the FZ [15], the economic impact will be devastating. In 2018, when some outbreaks in Brazil began to be detected, the Confederation of Agriculture and Livestock of Brazil (CNA) estimated an impact of R$ 1.3 to R$ 4.5 billion if the infection reached the free zone [12]. Thus, the intervention of the Official Veterinary Services (OVS), the productive sector, and the scientific community in the actions of control and eradication of CSF throughout the national territory is essential. Therefore, this work’s objective was to describe CSF’s history in Brazil, analyze the control and eradication actions, the recent outbreaks of CSFV infection in the NFZ, and the surveillance systems’ results in the FZ.

## 2. Epidemiological History of Classical Swine Fever in Brazil (1888 to 2001)

CSF was described in the world in the 19th century [7], and in Brazil, the first description of the disease dates back to 1888 in the states of Minas Gerais (MG) and São Paulo (SP) [12]. However, the first health legislation in which the CSF was cited, occurred in 1934, under Getúlio Vargas government, with the publication of Decree No. 24,548 of 31 July 1934, which approved the Regulation of the Animal Health Defense Service (DSA), a decree still in force in the country [16]. Article 61 of the order states that diseases such as swine erysipelas, also known as “red”, and swine fever, are subject to animal health protection measures. Since then, a sanitary work has been carried out, and the first national recognition of CSF FZ occurred in 2001 [17]. Still, the chronology of historical facts between 1888 and 2001 is described in Table 1.

In 1946, outbreaks of CSF in Rio de Janeiro, Minas Gerais, and São Paulo led to the death of approximately 2 million pigs directly by the disease, and many others due to the sacrifice determined by the health authorities. Therefore, from 1946 to 1951, the federal government implemented the first CSF Control Program [18]. 

In 1978, the first record of African swine fever (ASF) occurred in Brazil, in Rio de Janeiro, in pigs fed with food residues from international flights, this time from Portugal. Then, through presidential decree, the adoption of emergency measures to eradicate the disease in the country began [19]. The serological investigation for ASF control in Brazil started in 1980 and lasted until 1984. The most significant part of the samples collected comprised blood serum collected at the slaughterhouses with representative sampling from the municipalities and origin identification.

In this health emergency scenario due to ASF, the Ministry of Agriculture, Livestock and Food Supply (MAPA), in 1981, instituted the Program for Combating swine fever (PCSF) to eradicate ASF and control CSF [19]. In total, 288,369 serum samples were examined, resulting in 128 ASF positive samples (0.04%) during the four years of investigation. However, in the last three years of the research, no reagent samples were detected [20]. Brazil successfully established and applied the ASF eradication program, and in 1984, the infection was considered eradicated in the country.

After the eradication of ASF, CSF control became the main objective of the PCSF. The program was based on laboratory diagnosis, elimination of positive animals, vaccination against CSFV (Chinese strain), and serological monitoring in slaughterhouses. The actions were focused on identifying seronegative pigs and establishing free zones of the disease. Although this program had already been applied to control the infection, it was impossible to eliminate the virus from the national territory, and many outbreaks of CSF still occurred across the country. Due to the previous program’s failure, in 1992, the official program was reformulated to eradicate the virus, aiming at the progressive fight against the infection, prioritizing the regions based on the epidemiological conditions and the economic importance for the swine production [18].

The new program’s objective was the gradual removal of vaccination against CSFV across the country, and for this, laboratory diagnostic techniques were improved. Because of the further actions, the number of CSF outbreaks has decreased dramatically across the country, especially since the 1990s [18]. This reduction in outbreaks allowed the suspension of CSF vaccination in 1998, which was only carried out with the authorization and control from official services [18,21].

In 2000, a seroepidemiological survey was conducted based on scientific sampling methods to survey viral circulation in a wide area of the country, similar to the foot and mouth disease (FMD) free zone (FZ), enabling the use of the structure of existing official services. The results of this survey allowed MAPA, in 2001, to delimit and declare, based on evidence of the absence of viral circulation, a CSF FZ in Brazil [17]. This FZ was constituted by the states of Bahia (BA), Sergipe (SE), Espírito Santo (ES), Rio de Janeiro (RJ), Minas Gerais (MG), Tocantins (TO), Goiás (GO), Mato Grosso (MT), Mato Grosso do Sul (MS), São Paulo (SP), Paraná (PR), Santa Catarina (SC), Rio Grande do Sul (RS) and Distrito Federal (DF) [17].

The first CSF FZ in Brazil covered 51.7% of the national territory, 53.1% of the properties with swine, 78% of the pig herd, 87% of the housed breeding stock, and 93% of the slaughterhouses [18].

## 3. Implementation of Prevention and Surveillance Actions

The result obtained in the previous phase culminated in the national recognition of a CSF FZ [17]. Then, in 2004, the Normative Instruction No. 6 was made official for the entire national territory [22], establishing rules focused on CSF eradication and the Normative Instruction No. 27 [23], which instituted the contingency plan for CSF. Both regulations are still in effect.

CSF surveillance efforts were intensified with the publication of Internal Standards No. 5 in 2009 [24] and No. 3 in 2014 [25], which technically directed the actions’ systematization. The information produced by the active surveillance system for CSF supported the international recognition of the OIE’s Brazilian CSF FZ. It was widely used as a basis for international certification by Brazilian veterinary services to market pigs and their products.

Briefly, the components of the CSF surveillance system are described below:

1. Passive clinical surveillance from notification by owners, Official Veterinary Service or third parties;
2. Communication of the increase in mortality rates by qualified veterinarians assisting swine breeding establishments;
3. Active and continuous clinical surveillance in swine breeding establishments identified as having the highest risk of reintroducing CSF in the FZ;
4. Serological monitoring of intensive pig farming (IPF) by the collection of samples in slaughterhouses, from breeders, which were sent for disposal;
5. Seroepidemiological monitoring in non-technified pig herds (NTPig) at regular intervals;
6. Semiannual serological monitoring in Certified Swine Breeding Farms (CSBFs);
7. Ante and post-mortem inspection in swine slaughterhouses.

### 3.1. Surveillance in Wild Boar

If infected, populations of wild pigs may be the primary source for CSF introduction in domestic pig herds [26]. Due to the possible transmission of other infections to humans, domestic and native wild animals, especially wild pigs, were considered harmful in Brazil. Its management and control are regulated by the Brazilian Institute for the Environment and Renewable Natural Resources (IBAMA) [27].

The OIE Terrestrial Animal Health Code [9] recognizes the health status of a country, zone, or compartment regarding CSF by assessing some criteria relating to both populations: domestic and wild pigs. Among the standards adopted are the mandatory notification of CSF across the country and the encouragement of reports of clinical signs and cases compatible with the disease’s clinical manifestation. Therefore, if the government confirms that a surveillance program is implemented correctly, another OIE member country will not impose trade restrictions in response to the notification of the presence of the CSFV in the affected population. To this end, the Internal Standard DSA No. 3, from 18 September 2014 [25], mentions that in Brazilian states where domestic swine populations are considered to be CSF free, surveillance in wild pigs has a complementary function of validating the absence of infection. Then, the OVS of each state should updated data on the populations and habitat of the wild pigs. According to the implemented surveillance plan, the data obtained are intended to mitigate the risk that wild pigs may pose to domestic herds.

**Figure 1:**
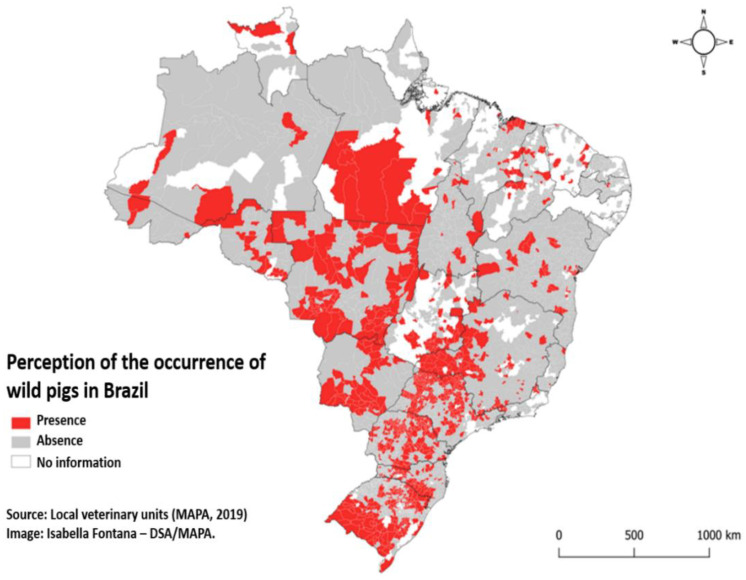
Perception of the occurrence of wild pigs in Brazil (Department of Animal Health of Ministry of Agriculture, Livestock, and Supply—Animal Health Defense Service (DSA)/Ministry of Agriculture, Livestock and Supply (MAPA), 2019 [13]).

In 2019, MAPA gathered information from the OVS to evaluate the perception of wild pigs in Brazil (Figure 1). The data are alarming, as these animals are found in all Brazilian biomes, demonstrating its high adaptability and dissemination capacity. Therefore, information on the occurrence and habitat of the wild pigs is essential for the surveillance program and the monitoring designs, which allows the generation of epidemiological data on the infection and the demonstration of free areas and possible interventions and containment measures for outbreaks. Besides, it is known that the health risk for the domestic swine populations is associated with the increase in the population density of the wild pigs, which is due both to the large number of hosts available and to the higher rate of contact between susceptible populations. Thus, the role of wild pigs in the maintenance of CSFV is of fundamental epidemiological importance.

The structuring of the epidemiological surveillance and population management program for wild pigs (Sus scrofa) in CSF FZ aims at the early detection of CSFV in wild pigs; detection of other important diseases; production of data to support the risk analysis processes in pig production; and assistance with the definition of health strategies for national pig production [25].

The strategies of the surveillance system for CSF in wild pigs include:

1. Passive surveillance based on reports of the presence of sick or dead animals, generating an epidemiological and clinical investigation with a collection of diagnostic material;
2. Active surveillance with an assessment of the biosecurity of farms, mapping the distribution of populations and definition of risk areas, analysis of data from the surveillance system, and collection of serological samples by voluntary management agents;
3. Serological surveillance carried out by fauna managers (hunters) who voluntarily adhere to the program. The legalized hunter must collect serological samples from these wild boars following biosafety measures to prevent the disease’s spread. To this end, the OVS, in partnership with Embrapa Swine and Poultry Research Center, has carried out various courses intending to train these partners, in addition to producing several instructive technical materials, such as the “Necropsy Manual for Swine” [28].

Thus, it is important that the abovementioned strategies are implemented correct and constantly checked to have specific data on the wild pig population distributed along the Brazilian Territory, but especially within the CSF free zone.

## 4. Expansion and Recognition of Classical Swine Fever Free Areas

In 2009, there was an expansion of national recognition of the CSF free area, which included the state of Rondônia (RO) [29]. Moreover, in 2013, there was the incorporation of the state of Acre (AC) and two municipalities in the state of Amazonas (AM), Guajará e Boca do Acre [30], accompanying the geographical composition and controls of FMD FZ with vaccination, recognized by the OIE.

Since 2014, the OIE has included the CSF in the list of the diseases evaluated by the Scientific Committee for Animal Disease, intending to recognize the health status of disease-free country or zone. Based on this, MAPA forwarded requests to the OIE for recognition of the CSF free zones, which were officially recognized in 2015 and 2016.

**Figure 2:**
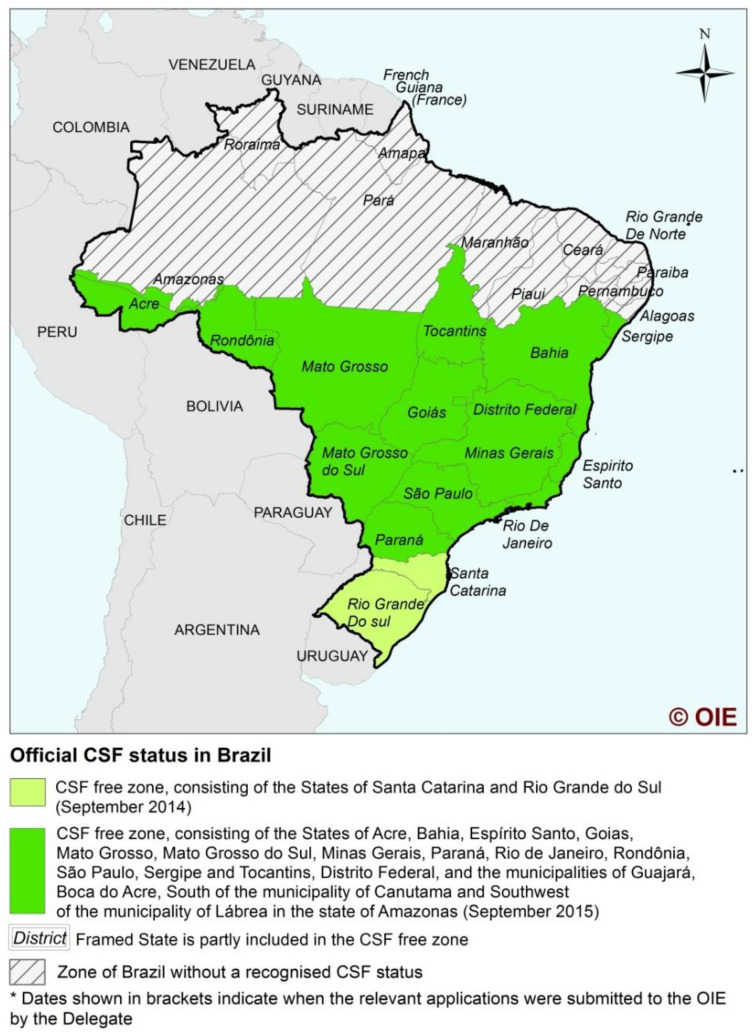
Classical swine fever-free zone in Brazil according to the World Organisation for Animal Health (OIE) [15].

This international recognition process prioritized the most relevant regions for producing and exporting pigs and their products. About 82% of the Brazilian pig herds are currently found in the CSF FZ, comprising approximately 50% of the national territory (Figure 2).

Brazil currently has a CSF NFZ formed by the States of Alagoas (AL), Amapá (AP), AM (except FZ region), Ceará (CE), Maranhão (MA), Pará (PA), Paraíba (PB), Pernambuco (PE), Piauí (PI), Rio Grande do Norte (RN) and Roraima (RR); and a FZ with recognition [8], divided in two certifications due to the claims being made in different years. The first was in 2015, including the states of SC and RS, and the second in 2016, when the states of AC, BA, DF, ES, GO, MT, MS, MG, PR, RJ, RO, SP, SE, TO, the municipalities of Guajará, Boca do Acre, south of the municipality of Canutama, and southwest of the municipality of Lábrea, belonging to the state of AM were included in the CSF FZ [31].

### 4.1. Health Status in the Classical Swine Fever Non-Free Zone

The NFZ covers about 50% of the Brazilian territory, comprising approximately 18% of the national pig herd, distributed in more than 300 thousand rural establishments, predominantly made up of small family farmers. The NFZ includes 11 Brazilian states (AM, RR, PA, AP, MA, PI, CE, RN, PB, PE and AL), that present diverse productive, social, economic, and environmental characteristics, characteristics that difficult the development of necessary actions to control and eradicate CSF. Although in smaller numbers, this region also has farms with technified production that will benefit directly from reducing the risk of occurrence of CSF and the end of restrictions on trading their products [12].

Pig production in this region represents an essential source of income and animal protein for local populations, especially for small rural producers in a situation of socio-economic vulnerability, who practice, in their majority, the extensive pig production. In this production system, the animals are raised free, without more significant concerns on productivity. Animals of different categories are raised and handled jointly, making health or zoo technical control difficult [32,33].

As observed in the 1990s, the CSF outbreaks declined significantly in the NFZ. In CE, which is located in the Northeast of Brazil, 12 outbreaks were reported in 2001, zero in 2002, four in 2003, and one in 2004 [18]. Between 2005 and 2017, several outbreaks occurred in CE and in the other five states in the North and Northeast regions of Brazil (Table S1). In this period, the outbreaks occurred between February and August, coinciding with the highest occurrence described by Krzyzaniak in a study carried out in the State of São Paulo from 1990 to 1996 [34].

#### 4.1.1. Identification of New Outbreaks of Classical Swine Fever in the Non-Free Zone (2018 to 2019)

**Figure 3:**
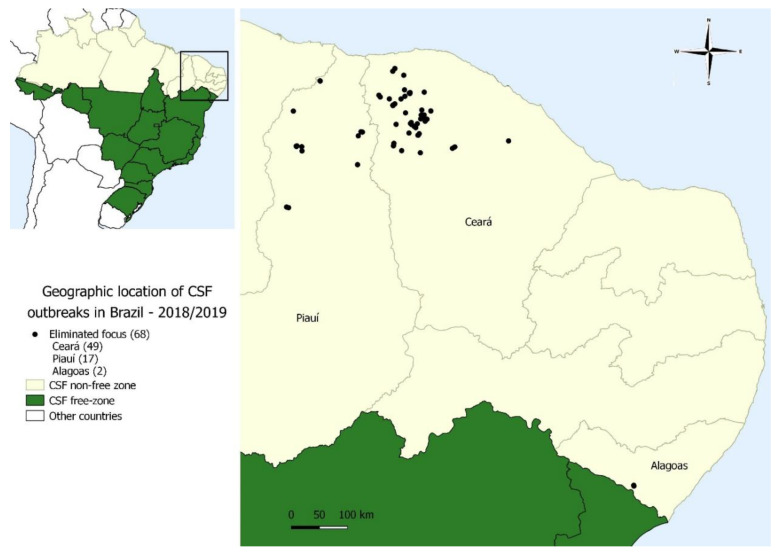
Geographic location of classical swine fever outbreaks in the period from 2018 to 2019 in Brazil (DSA/MAPA, 2019).

After improving the surveillance system in the NFZ, in August 2018, new cases of clinical CSF cases were confirmed in the state of CE. Since then, 68 outbreaks of the clinical disease have been recorded (data until February 2020) in 28 municipalities in Brazil’s Northeast region. The outbreaks emerged in 20 cities in the state of CE, seven cities in the state of PI, and one focus in the state of AL (Table 2; Table S2). However, it is important to mention that all outbreaks occurred in the subsistence pig system. Animals are in close contact with those from other properties in the same category, without specific biosafety measures (Table 2, Figure 3).

During the emergence of the outbreaks, all CSF cases were identified, either through notifications to OVS, regarding animals presenting clinical signs that were suggestive of infection, or through OVS active surveillance within or near previously reported outbreak areas. Because it is an NFZ, the containment measures regulated by the Normative Instruction No. 27 [23] have not been fully applied. Nevertheless, complementary investigations prioritized the surveillance zone and epidemiological links; therefore, all suspicious farms in the region were visited, and all animals in the confirmed outbreaks were eliminated [35]. The states of CE, PI, and AL, where the last CSFV outbreaks in Brazil were notified, are part of the NFZ, in which the condition of infection-free was never declared. Besides, it is essential to consider some restrictions on animals’ movement and their by-products between the CSF NFZ and FZ [35].

The first outbreak was detected in the municipality of Forquilha (CE), in August 2018, by the surveillance actions for the swine hemorrhagic disease syndromes carried out by ADAGRI—Agricultural Defense Agency of the state of CE. However, laboratory analysis confirmed the CSF outbreak two months later, in October 2018 (Table 2). This focus was located more than 500 km from the limits of the FZ.

An intensification on surveillance occurred within the NFZ since the first outbreak in CE, which enabled the detection of new suspicious cases, confirming another 67 new CSF outbreaks. In October 2019, outbreaks were also detected in the state of PI, in which the focus was more than 300 km away from the border limits of the FZ. Still, new outbreaks in subsistence creation were identified in the state of AL practically one year after the occurrence in the municipality of Forquilha (CE) [35].

To completely eradicate the CSFV from Brazilian territory, the “Brazil CSF-Free Strategic Plan” is being implemented in this NFZ zone. Moreover, the Brazilian government has been intensifying the actions to strengthen the CSF’s NFZ OVS, including training professionals in a veterinary emergency, improving the surveillance system, property re-registration, and animal health education. During the outbreaks, the OVS’s health actions, mainly associated with epidemiological, resulted in the investigation of approximately 2457 properties during the 68 outbreaks in three states (Table 3).

Since the first notification of the disease near the FZ in 2018, the OVS has reinforced the surveillance and prevention actions, inspecting pigs’ traffic and its by-products. It occurred mainly in the states bordering the NFZ, aiming at increasing the protection of the CSF FZ of Brazil.

After notification of outbreaks in the CSF NFZ, the OVS has also intensified clinical surveillance on FZ properties. In the state of SE, bordering the state of AL, 1006 properties and approximately 19,000 pigs were inspected and showed no clinical signs of hemorrhagic disease. Likewise, in the state of BA, which borders the states PI, PE, SE, and AL, 4290 properties were inspected, with no evidence of CSFV infection in this area. The monitoring system for swine hemorrhagic diseases in the NFZ is revised continuously to better adapt to the organizational conditions of pig production in the states located in that region, aiming to establish a more effective control/eradication program. 

The actions adopted in the outbreaks were conducted as recommended by the OIE Terrestrial Animal Health Code [9] and the contingency plan for CSF [23]. These actions were related to traffic control within the country; surveillance outside of the containment and/or protection zone; surveillance within the containment and/or protection zone; traceability; official destruction of products of animal origin; official disposal of carcasses, by-products, and waste; zoning; disinfection; prohibited vaccination and no treatment for affected animals [35].

Regarding traffic control between the NFZ and the FZ after recognition by the OIE (2015 and 2016), Brazil had already adopted official measures to restrict traffic between zones with different statuses. The last update of the legislation took place in the form of Normative Instruction No. 25 of 19 July 2016, which imposed other traffic restrictions to enhance the previous actions [31]. Most measures were applied through fixed posts and mobile inspection teams strategically located within the limits of the FZ, considering existing natural and perennial barriers, such as rivers, mountain-chains, and native forests.

Furthermore, the disease has been causing significant social and economic impacts and concerns about the possible reintroduction of CSFV in the Brazilian FZ. It is known that until the end of July 2019, the OVS operational costs and the costs related to the compensation to producers exceeded the value of R$1.5 million [12]. However, with the implementation of other measures proposed by the contingency plan for CSF [22], it is expected a benefit of R$394 million in the 15 years.

#### 4.1.2. Challenges in Controlling Outbreaks of Classical Swine Fever in the Non-Free Zone of Brazil

Despite the adoption of health measures to eliminate the outbreaks, the actions were not sufficient to reduce or eliminate the disease’s occurrence due to several factors. The region has diverse, productive, social, economic, and environmental characteristics, which increases the challenge for the development of actions necessary to control and eradicate CSF. Pig production in this region represents an important source of income and animal protein for local populations, especially for small rural producers in a situation of socio-economic vulnerability, who practice, in their majority, the production of pigs extensively [12,33]. 

The productive, social, economic, and environmental characteristics increase the challenge of developing the actions necessary to control and eradicate CSF. Therefore, knowing and analyzing aspects of local pig production helps technicians understand health problems, providing support for developing specific strategies to face the challenge. A precise situational diagnosis, including a detailed characterization of the production’s conditions and commercial relations, is essential for effective planning and the direction and management of CSF eradication actions.

The quality of the information available is a necessary subsidy for planning and implementing intervention actions. The deficiency in coverage and updating of pig creations is recognized, especially in subsistence production, where the variation between the data is quite expressive [12]. Therefore, this scenario points to a clear need to invest resources, technology, and time to characterize the productive system, as the registration and maintenance of cadastral data and animal population seem to be quite unstable in this region, reinforcing the importance of the contingency plan for CSF [23].

Another critical factor, notably in the states of the Northeast Region of Brazil, is the commercialization of live pigs in open markets with other animal aggregations, with the participation of intermediaries [12,36]. They acquire pigs in different properties, mixing different origins, for sale at fairs that distribute animals to various destinations. This characteristic of animal movement represents a significant risk factor and must be considered in the region’s strategies for health evolution. This area also has a few swine slaughterhouses with official inspection [37], which reinforces the importance of CSF surveillance to decrease the places of illegal slaughter of pigs, characterized as risk points, and therefore, requiring special attention by the OVS.

The lack of passive surveillance activities for swine hemorrhagic syndrome, with the failure of several states in registering these occurrences’ suspicions, contributes to the lack of acknowledgement of the epidemiological situation of the infection [12,38]. One of the main factors for the success of any intervention strategy against CSF in the CSF NFZ region is the strengthening of OVSs [12], which demands investments to enable the execution of animal health defense actions.

As a complement to the CSF control measures in the NFZ, by the Normative Instruction No. 10 of 6 April 2020, the use of the CSFV vaccine in the CSF NFZ was authorized, following the Brazil CSF-Free Strategic Plan approved by Ordinance DSA No. 264 of 10 December 2019 [12]. Still, in endemic regions with domestic pigs and with little international trade, the main goal is to protect against losses due to clinical disease, and gradually reduce CSF occurrence, leading to the eradication of the disease [6]. Before starting vaccination in each state of the NFZ, the Animal Health Department (DSA) will carry out a specific assessment of the implementation of the CSF-Free Brazil Strategic Plan. It is still conditioned that the traffic of vaccinated pigs in the NFZ will take place under particular conditions, approved in complementary acts of the DSA. Article 4 of this Normative Instruction came into effect on 4 May 2020 [12].

#### 4.1.3. Health Surveillance System in the Classical Swine Fever-Free Zone

In Brazil, CSF is a disease of mandatory immediate notification to the OVS, which maintains a surveillance system for pigs’ hemorrhagic disease. This system is established by federal regulations aimed at supporting the FZ status of CSF, preventing the reintroduction of the virus, and ensuring the early detection and prompt response to all suspected cases of CSF. To this end, permanent, systematic surveillance actions and standardized strategies are carried out for the entire pig production chain and the elimination and eradication of any outbreaks of the disease in the NFZ. It is important to emphasize that the last occurrence of CSF in the FZ was in January 1998, in the state of São Paulo [12].

The surveillance system for CSF in Brazilian FZ is also based on the actions described above in Section 3 and Section 3.1. The blood serum samples submitted to surveillance are screened for antibodies against CSFV by the enzyme-linked immunosorbent assay (ELISA). Then, the reagent samples are, in turn, subjected to confirmation, using the virus neutralization (VN) and PCR techniques. During the years 2015 to 2019, serological surveys were conducted as part of the surveillance actions for CSF and are summarized in Table 4.

As part of the actions to maintain the Brazilian CSF FZ status, active and passive surveillance actions were carried out between 1 July 2018, and 30 June 2019, and submitted to the OIE in 2019 to prove the infection-free status in the FZ. During this period, 23,647 commercial and subsistence pigs were inspected by the OVS, and no hemorrhagic disease was found. In the same period, 662 notifications of increased mortality in pigs registered by the OVS were investigated, refuting clinical, epidemiological, or laboratory suspicions of CSF.

During the abovementioned period, 78,134 blood serum samples from active serological surveillance in domestic pigs were submitted to ELISA. Of those, 32 samples from 30 herds were reactive, with the suspicion of CSFV being ruled out after negative results on VN and PCR tests, in conjunction with a clinical, epidemiological investigation, confirming the absence of CSFV circulation in the area. Besides, ante- and post-mortem inspections were carried out on 41,867,018 pigs in slaughterhouses, with no clinical signs compatible with CSF. Regarding the surveillance in the wild pigs’ populations carried out by the OVS, 566 blood serum samples were tested for CSFV using ELISA. Of these, 565 samples were negative for CSFV, and only one sample was reagent. Again, in the following complementary molecular and epidemiological investigations, the occurrence of CSF was discarded.

In October 2019, an active serological surveillance survey was conducted in subsistence creations located in neighboring international municipalities and CSF NFZ (states of AC, BA, MS, MT, RO, SE, and TO) of Brazil. The results showed an absence of CSFV circulation, agreeing with the information from active surveillance system for CSF used as basis for international certification by veterinary services for the trade of pigs and their products, originated in the FZ. Still, the control of live pigs’ imports, genetic material, and pig products follows the recommendations of the OIE Terrestrial Animal Health Code, and therefore, the traffic of pigs and by-products from NFZ (where outbreaks of CSF have been confirmed since October 2018) to FZ is prohibited. 

Based on the previous information, Brazil declares that the CSF FZ’s epidemiological situation remains unchanged, maintaining the CSF free zone status with the OIE.

## 5. Final Considerations

The absence of CSF outbreaks in the FZ, since 1998, is a strong indication that the surveillance systems for CSF, together with the control and eradication measures implemented in the FZ, have been effective in controlling the disease. However, the existence of a NFZ within the country and the recent occurrence of outbreaks in the 2018–2019 biennium highlight the potential risk of reintroducing the FZ disease. It is important to highlight that this review includes CSF surveillance data of up to 20 July 2020 and does not include information regarding outbreaks beyond this timepoint.

Countries with large territorial extensions and diverse economic realities face greater impasses regarding CSF since control actions are affected by other issues such as regional differences in infrastructure, presence of technically skilled labor, and availability of financial resources. Still, the uneven development of the territories creates situations in which intensive and industrial herds coexist in the same national territory with subsistence pig creations, where animals are raised in a semi-extensive way [39]. 

In Brazil, states where pig farming is predominantly industrial, and the production substantially participates in the development of the economy, also reported better conditions for the production of epidemiological data, application of prophylactic measures, and compliance with standards possible to monitor the health status of herds. However, in the territories of the NFZ, human development rates are lower in some areas. Non-technified pig herds are predominant, with low participation in the local economy and focusing on the production and fattening of animals, relegating the health focus to the background [40]. 

Non-technified pig herds have an important social role as an income and feed generator in poor regions where family farming prevails. Still, they are often exposed to severe health problems due to poor infrastructure, low biosafety levels, and lack of regular veterinary medical care [41]. Besides, it is common that in regions where this type of production is predominantly practiced, difficulties control CSF occurrence, even if there is an active surveillance system [41]. Therefore, the socio-economic context in these regions should be considered since it may disfavor the control and eradication of the CSFV infection. 

Another complicating factor resides in the difficulty of controlling CSF in large territorial extensions. This is mainly due to problems in the effective inspection in traffic of animals, products, and by-products from the NFZ to the FZ, which could reintroduce the CSFV in industrial pig herds. Since the circulation of infected animals or even workers involved in pig farming between disease-free zones and outbreaks was identified as a possible risk factor for introducing the disease [42], effective programs, including measures for different situations, are needed. In fact, the punishment proportional to the risk in cases of non-compliance with rules should be considered as well.

To this end, active and passive health surveillance actions should continue to be fully applied in the CSF FZ to prevent the reintroduction of the disease and investments and studies to adapt the activities to the productive reality of the NFZ, especially in the border regions. Moreover, further studies on the NFZ aiming to generate data on CSF’s real situation in the regional pig productions are necessary for structuring effective actions, even though the local reality presents possible difficulties for executing such work. Besides, new outbreaks could be avoided by adopting diagnostic techniques based on biological samples that facilitate surveillance activities, such as oral fluid. A recent study showed that the detection of CSFV by PCR in oral fluids showed exciting results in the field, as well as resulting in a 23–40% reduction in the number of sampled animals (samples were collected by pen, as a pool) and consequently, in the use of materials [43]. Still, the development of vaccines capable of differentiating the immune response of vaccinated and infected animals could be of great help to guarantee disease control in the non-free zone.

Finally, the structuring of a contingency plan for wild pigs, the construction of co-responsibility strategies with the productive sector, as the biosecurity of farms and creating an indemnity fund, are essential factors that must be considered in the control of the disease and the consequent regional socio-economic impact.

## 6. Conclusions

CSF control and eradication activities in most of the Brazilian territory can be considered successful, taking into account that the last CSF outbreak reported in the FZ occurred in 1998 in the state of SP. This fact indicates the robust surveillance systems for CSF in the FZ. From that period, the free status was granted by the OIE to an extensive area that comprises approximately 95% of the Brazilian industrial swine production. However, outbreaks in parts of the territory characterized as CSF NFZ highlight the potential risk of reintroducing the disease in the FZ. Therefore, considering the epidemiological, productive, and social characteristics of the NFZ, it is essential to intensify surveillance and to carry on the disease control and eradication program. Likewise, localities bordering the FZ need to be prioritized to avoid the reintroduction of CSF in this area, which would negatively affect the pig production chain in Brazil, not only in the health aspect but also regarding international trade.

## Figures and Tables

**Table 1:** Chronological facts regarding classical swine fever and African swine fever in Brazil.

Year	Facts
1888	First detection of classical swine fever in the states of Minas Gerais and São Paulo
1934	Approval of the Regulation of the Animal Health Defense Service by Decree No. 24548 of 3 July 1934
1946–1951	First official animal health program that instituted classical swine fever control
1978	First report of African swine fever
1981	Program for combating swine fever (PCSF) was established
1984	African swine fever eradication
1992	Approval of the Classical Swine Fever Control and Eradication Program (PCECSF) and withdrawal of vaccination in the South region
1998	Suspension of vaccination against classical swine fever throughout the country
2001	Declaration of classical swine fever-Free Zone by the Ministry of Agriculture, Livestock and Supply (MAPA) by Normative Instruction No. 01 of 4 January 2001.

**Table 2:** Classical swine fever outbreaks in the non-free zone from 2018 to 2019 in Brazil.

Year	Outbreak	Start	Resolved	City	State	Susceptible	Cases	Deaths	Killed/Disposed of	Percent Rate			Laboratory Test *
										Apparent Morbidity	Apparent Mortality	Apparent Case Fatality	
	1	5 August 2018	11 October 2018	Forquilha	Ceará	44	25	21	23	56.82	47.73	84.00	+
2018	2	5 August 2018	18 October 2018	Varjota ^1^	Ceará	21	21	15	06	100.00	71.43	71.43	+
	3	7 August 2018	17 October 2018	Groaíras	Ceará	77	32	27	50	41.56	35.06	84.38	+
	4	25 August 2018	11 October 2018	Forquilha ^2^	Ceará	132	116	112	20	87.88	84.85	96.55	+
	5	16 August 2018	5 November 2018	Moraújo ^3^	Ceará	227	39	36	191	17.18	15.86	92.31	+
	6	2 September 2018	9 November 2018	Frecheirinha	Ceará	10	06	06	04	60.00	60.00	100.00	+
	7	12 September 2018	18 October 2018	Varjota ^4^	Ceará	08	08	04	04	100.00	50.00	50.00	+
	8	15 September 2018	23 November 2018	Cariré	Ceará	26	24	15	11	92.31	57.69	62.50	+
	9	30 September 2018	31 October 2018	Cariré	Ceará	21	05	04	17	23.81	19.05	80.00	+
	10	3 October 2018	15 November 2018	Ipu	Ceará	216	50	20	196	23.15	9.26	40.00	+
	11	9 October 2018	16 October 2018	Santa Quitéria	Ceará	14	04	02	12	28.57	14.29	50.00	+
	12	9 October 2018	21 October 2018	Santa Quitéria	Ceará	03	01	01	02	33.33	33.33	100.00	+
	13	9 October 2018	26 October 2018	Reriutaba	Ceará	19	19	06	13	100.00	31.58	31.58	+
	14	12 October 2018	26 October 2018	Cariré	Ceará	22	07	07	15	31.82	31.82	100.00	+
	15	13 October 2018	23 October 2018	Groaíras	Ceará	107	67	67	40	62.62	62.62	100.00	+
	16	14 October 2018	13 November 2018	Mulungu	Ceará	18	18	06	12	100.00	33.33	33.33	+
	17	15 October 2018	18 October 2018	Varjota ^5^	Ceará	26	02	0	26	7.69	0.00	0.00	+
	18	15 October 2018	9 November 2018	Cariré	Ceará	34	04	01	33	11.76	2.94	25.00	+
	19	20 October 2018	5 February 2019	Moraújo	Ceará	163	28	26	137	17.18	15.95	92.86	+
	20	22 October 2018	1 November 2018	Graça	Ceará	08	08	03	05	100.00	37.50	37.50	+
	21	23 October 2018	17 November 2018	Hidrolândia	Ceará	80	25	24	56	31.25	30.00	96.00	+
	22	24 October 2018	17 December 2018	Reriutaba	Ceará	128	12	06	122	9.38	4.69	50.00	+
	23	25 October 2018	23 Janurary 2019	Reriutaba	Ceará	10	07	07	03	70.00	70.00	100.00	+
	24	25 October 2018	27 November 2018	Martinópole	Ceará	20	12	12	08	60.00	60.00	100.00	+
	25	28 October 2018	23 Janurary 2019	Reriutaba	Ceará	176	25	16	160	14.20	9.09	64.00	+
	26	31 October 2018	1 December 2018	Frecheirinha	Ceará	193	56	36	157	29.02	18.65	64.29	+
	27	1 November 2018	9 November 2018	Frecheirinha	Ceará	41	22	19	22	53.66	46.34	86.36	+
	28	3 October 2018	30 Janurary 2019	Groaíras	Ceará	227	41	40	187	18.06	17.62	97.56	+
	29	5 November 2018	29 Janurary 2019	Groaíras	Ceará	105	09	07	98	8.57	6.67	77.78	+
	30	7 November 2018	3 December 2018	Moraújo	Ceará	39	05	04	35	12.82	10.26	80.00	+
	31	10 November 2018	28 November 2018	Groaíras	Ceará	06	02	02	04	33.33	33.33	100.00	+
	32	10 November 2018	29 Janurary 2019	Cariré	Ceará	91	22	06	85	24.18	6.59	27.27	+
	33	17 November 2018	30 Janurary 2019	Tianguá	Ceará	159	36	29	130	22.64	18.24	80.56	+
	34	18 November 2018	21 December 2018	Moraújo	Ceará	305	13	12	293	4.26	3.93	92.31	+
	35	19 November 2018	4 December 2018	Tianguá	Ceará	43	03	01	42	6.98	2.33	33.33	+
	36	20 November 2018	8 February 2019	Coreaú	Ceará	329	07	02	327	2.13	0.61	28.57	+
	37	22 November 2018	7 February 2019	Sobral	Ceará	57	28	23	34	49.12	40.35	82.14	+
	38	12 December 2018	24 Janurary 2019	Groaíras	Ceará	02	02	01	01	100.00	50.00	50.00	+
2019	39	5 Janurary 2019	8 February 2019	Croatá	Ceará	26	24	21	05	92.31	80.77	87.50	+
	40	25 Janurary 2019	16 May 2019	Cariré ^6^	Ceará	153	61	13	140	39.87	8.50	21.31	+
	41	1 February 2019	11 June 2019	Viçosa do Ceará	Ceará	54	54	50	04	100.00	92.59	92.59	+
	42	9 February 2019	12 March 2019	Granja	Ceará	22	21	04	18	95.45	18.18	19.05	+
	43	10 February 2019	18 May 2019	Cabeceiras do Piauí	Piauí	32	20	20	12	62.50	62.50	100.00	+
	44	12 February 2019	12 March 2019	Granja	Ceará	20	20	13	07	100.00	65.00	65.00	+
	45	28 February 2019	27 March 2019	Croatá	Ceará	02	02	0	02	100.00	0.00	0.00	+
	46	1 March 2019	8 May 2019	Murici dos Portelas	Piauí	35	26	26	09	74.29	74.29	100.00	+
	47	4 March 2019	7 June 2019	Granja ^6^	Ceará	26	04	03	23	15.38	11.54	75.00	+
	48	7 March 2019	7 April 2019	Lagoa do Piauí	Piauí	13	08	07	06	61.54	53.85	87.50	+
	49	8 March 2019	17 May 2019	Cariré	Ceará	155	84	71	84	54.19	45.81	84.52	+
	50	10 March 2019	31 May 2019	Brasileira	Piauí	37	06	04	33	16.22	10.81	66.67	+
	51	10 March 2019	11 May 2019	Cabeceiras do Piauí	Piauí	30	22	21	09	73.33	70.00	95.45	+
	52	29 March 2019	10 May 2019	Cabeceiras do Piauí	Piauí	37	07	02	35	18.92	5.41	28.57	+
	53	30 March 2019	8 May 2019	Murici dos Portelas	Piauí	12	12	0	12	100.00	0.00	0.00	+
	54	1 April 2019	31 May 2019	Brasileira	Piauí	40	29	28	12	72.50	70.00	96.55	+
	55	5 April 2019	5 May 2019	Cabeceiras do Piauí	Piauí	48	01	0	48	2.08	0.00	0.00	+
	56	8 April 2019	13 April 2019	Lagoa do Piauí	Piauí	16	02	01	15	12.50	6.25	50.00	+
	57	10 April 2019	8 May 2019	Murici dos Portelas	Piauí	12	01	0	12	8.33	0.00	0.00	+
	58	12 April 2019	8 May 2019	Murici dos Portelas	Piauí	19	01	0	19	5.26	0.00	0.00	+
	59	13 April 2019	27 July 2019	Massapê	Ceará	34	21	21	13	61.76	61.76	100.00	+
	60	22 April 2019	18 May 2019	Cabeceiras do Piauí	Piauí	22	14	13	09	63.64	59.09	92.86	+
	61	27 April 2019	18 May 2019	Domingos Mourão	Piauí	41	13	12	29	31.71	29.27	92.31	+
	62	21 May 2019	10 June 2019	Milton Brandão	Piauí	26	08	04	22	30.77	15.38	50.00	+
	63	25 May 2019	5 July 2019	São João do Arraial	Piauí	181	108	98	83	59.67	54.14	90.74	+
	64	19 July 2019	23 August 2019	Viçosa do Ceará	Ceará	10	10	05	05	100.00	50.00	50.00	+
	65	23 August 2019	18 October 2019	Coreaú ^6^	Ceará	259	22	20	239	8.49	7.72	90.91	+
	66	7 September 2019	9 October 2019	Traipu	Alagoas	32	32	02	30	100.00	6.25	6.25	+
	67	1 October 2019	15 October 2019	Traipu	Alagoas	06	04	0	06	66.67	0.00	0.00	+
	68	5 October 2019	12 November 2019	Domingos Mourão ^6^	Piauí	29	11	10	19	37.93	34.48	90.91	+
Total	68	-	-	-	-	4636	1459	1095	3541	31.47	23.62	75.05	-

Adapted: OIE [35]. * Positive by real-time reverse transcriptase/polymerase chain reaction (rRT-PCR), virus isolation (VI) on cell culture or virus neutralization test (VNT) and performed by the Federal Livestock and Agriculture Laboratory in Minas Gerais (LFDA/Minas Gerais (MG)) or Federal Livestock and Agriculture Laboratory in Pernambuco (LFDA/Pernambuco (PE)) both National laboratories in Brazil; ^1^ Four pigs were sold to another property in the same municipality, confirmed as an outbreak, and locally slaughtered. ^2^ Population data has been updated according to complementary investigations. ^3^ This focus was constituted by several subsistence properties, forming a sizeable epidemiological unit. ^4^ Three pigs were sold to another feature in the same municipality, confirmed as an outbreak, and locally slaughtered. ^5^ Property with an epidemiological link to two other outbreaks in the same municipality after receiving seven pigs from the two outbreaks. ^6^ The total number of susceptible animals, cases, deaths, and animals killed and eliminated were modified after further investigation. All contact pigs were considered to belong to the same epidemiological unit and were eliminated.

**Table 3:** Reports’ summary of the Official Veterinary Service during the outbreaks of classical swine fever in 2018 and 2019, by state.

Actions of OVS *	States			Total
	Ceará	Piauí	Alagoas	
Notified outbreaks	49	17	2	68
Investigated properties	1100	946	411	2457

* Official Veterinary Services.

**Table 4:** Seroepidemiological surveys for classical swine fever carried out between 2015 and 2019, in Brazil, in the free zone.

Year	Certified Pig Breeding Farm		Intensive Pig Farming		Discard Breeders		Wild Pigs	
	Samples	ELISA *-Positive (%)	Samples	ELISA-positive (%)	Samples	ELISA-Positive (%)	Samples	ELISA-Positive (%)
2015	50,536	6 (0.01)	13,965	0 (0.00)	15,092	4 (0.03)	-	-
2016	44,808	9 (0.02)	8,818	0 (0.00)	23,362	13 (0.05)	57	0 (0.00)
2017	42,229	26 (0.06)	11,351	9 (0.08)	20,600	7 (0.03)	315	0 (0.00)
2018	40,963	6 (0.01)	19,757	8 (0.04)	22,277	11 (0.05)	642	1 (0.15)
2019	41,669	5 (0.01)	17,798	7 (0.04)	25,447	29 (0.11)	594	0 (0.00)
**Total**	220,205	55 (0.02)	71,689	24 (0.03)	106,778	64 (0.06)	1608	1 (0.06)

* ELISA: enzyme-linked immunosorbent assay.

## Supplementary Materials

The following are available online at https://www.mdpi.com/1999-4915/12/11/1327/s1, Table S1: outbreaks of classical swine fever in the non-free zone from 2005 to 2017 in Brazil. Table S2: geographic location of classical swine fever outbreaks in the non-free zone between 2018 and 2019 in Brazil.
