# Supplementary material for: Achievements and Challenges of Classical Swine Fever Eradication in Brazil
Source: Viruses. 2020 Nov 19;12(11):1327. doi: 10.3390/v12111327 (PMC7699269; doi:10.3390/v12111327)
Supplement: Supplementary file 1 [file viruses-12-01327-s001.pdf]

Table S1. Outbreaks of classical swine fever in the non-free zone from 2005 to 2017 in Brazil.

Year	Outbreak	Start	Resolved	City	State	Susceptible	Cases	Deaths	Percent rate			Test**
									Apparent morbidity	Apparent mortality	Apparent case lethality	
2005	NC*	-	-	-	-	-	-	-	-	-	-	-
2006	1	22/02/2006	02/06/2006	Cuitegi	Paraíba	170	66	49	38.82	28.82	74.24	+
	2	21/03/2006	02/06/2006	Tianguá	Ceará	155	45	45	29.03	29.03	100.00	+
	3	09/04/2006	02/06/2006	Tianguá	Ceará	26	04	03	15.38	11.54	75.00	+
	4	26/04/2006	02/06/2006	Sobral	Ceará	44	03	03	6.82	6.82	100.00	+
	5	05/05/2006	02/06/2006	Tianguá	Ceará	02	01	01	50.00	50.00	100.00	+
	6	05/05/2006	02/06/2006	Coreaú	Ceará	103	61	56	59.22	54.37	91.80	+
	7	26/05/2006	02/06/2006	Ibiapina	Ceará	10	01	01	10.00	10.00	100.00	+
	8	02/06/2006	02/06/2006	Caucaia	Ceará	172	05	05	2.91	2.91	100.00	+
2007	1	16/01/2007	24/04/2007	Caucaia	Ceará	41	03	03	7.32	7.32	100.00	+
2008	1	14/08/2008	27/09/2008	Barra do Corda	Maranhão	35	12	12	34.29	34.29	100.00	+
2009	1	21/02/2009	26/03/2009	Mossoró	Rio Grande do Norte	53	24	22	45.28	41.51	91.67	+
	2	14/03/2009	02/04/2009	Jucurutu	Rio Grande do Norte	73	36	36	49.31	49.31	100.00	+
	3	22/03/2009	03/04/2009	Jucurutu	Rio Grande do Norte	44	14	13	31.82	29.54	92.86	+
	4	23/03/2009	03/04/2009	Mossoró	Rio Grande do Norte	55	02	01	3.64	1.82	50.00	+
	5	04/04/2009	16/04/2009	Jucurutu	Rio Grande do Norte	14	04	03	28.57	21.43	75.00	+
	6	07/04/2009	15/04/2009	Jucurutu	Rio Grande do Norte	11	01	01	9.09	9.09	100.00	+
	7	15/04/2009	26/04/2009	Mossoró	Rio Grande do Norte	68	34	24	50.00	35.29	70.59	+
	8	22/04/2009	01/05/2009	Mossoró	Rio Grande do Norte	47	41	39	87.23	82.98	95.12	+
	9	18/05/2009	09/06/2009	Jucurutu	Rio Grande do Norte	30	17	13	56.67	43.33	76.47	+
	10	05/05/2009	20/06/2009	Macapá	Amapá	449	293	293	65.26	65.26	100.00	+
	11	25/05/2009	18/06/2009	Jucurutu	Rio Grande do Norte	20	17	14	85.00	70.00	82.35	+
	12	03/06/2009	17/07/2009	Macaíba	Rio Grande do Norte	301	74	74	24.58	24.58	100.00	+
	13	04/06/2009	01/07/2009	Macaíba	Rio Grande do Norte	120	120	118	100.00	98.33	98.33	+
	14	07/06/2009	17/06/2009	Macapá	Amapá	04	02	02	50.00	50.00	100.00	+
	15	08/06/2009	21/07/2009	Ilha de Marajó	Pará	44	01	0	2.27	0.00	0.00	+
	16	08/06/2009	21/07/2009	Ilha de Marajó	Pará	200	04	0	2.00	0.00	0.00	+
	17	10/06/2009	20/08/2009	Macapá	Amapá	60	06	06	10.00	10.00	100.00	+
	18	11/06/2009	20/06/2009	Macapá	Amapá	18	03	03	16.67	16.67	100.00	+
2010-2017	NC	-	-	-	-	-	-	-	-	-	-	-
**Total**	**28**	**-**	**-**	**-**	**-**	**2,369**	**894**	**840**	**37.74**	**35.46**	**93.96**	**-**

Adapted: OIE [35].

*NC: No cases have been reported during this period.

***Positive by virus isolation on cell culture performed in the Agriculture and Livestock National Laboratory (LANAGRO-PE), Recife, Pernambuco (National laboratory), Brazil.*

Table S2. Geographic location of classical swine fever outbreaks in the non-free zone between 2018 and 2019 in Brazil.

Outbreaks	Location	State	Latitude (Degrees/ Minutes/ Seconds)	Longitude Degrees/ Minutes/ Seconds)	Latitude (Decimal Degrees)	Longitude (Decimal Degrees)
1	Forquilha	Ceará	-3 48 34,5	-40 15 19,38	-3,809583333	-40,25538333
2	Varjota	Ceará	-4 12 16,81	-40 27 38,86	-4,204669444	-40,46079444
3	Groaíras	Ceará	-3 56 39,1	-40 19 3,8	-3,944194444	-40,31772222
4	Forquilha	Ceará	-3 48 33,41	-40 15 21,44	-3,809280556	-40,25595556
5	Moraújo	Ceará	-4 17 53,9	-38 59 22,4	-4,298305556	-38,98955556
6	Frecheirinha	Ceará	-3 30 33,98	-40 35 33,15	-3,509438889	-40,59254167
7	Varjota	Ceará	-4 12 15,52	-40 27 58,19	-4,204311111	-40,46616389
8	Cariré	Ceará	-4 29 27,1	-40 25 33,5	-4,490861111	-40,42597222
9	Cariré	Ceará	-4 1 38,3	-40 49 15,8	-4,027305556	-40,82105556
10	Ipu	Ceará	-3 13 42	-40 41 45,9	-3,228333333	-40,69608333
11	Santa Quitéria	Ceará	-4 25 6,5	-39 54 13,5	-4,418472222	-39,90375
12	Santa Quitéria	Ceará	-4 23 42,7	-39 51 54,5	-4,395194444	-39,86513889
13	Reriutaba	Ceará	-4 10 49,69	-40 26 40,85	-4,180469444	-40,44468056
14	Cariré	Ceará	-3 55 19	-40 22 41,8	-3,921944444	-40,37827778
15	Groaíras	Ceará	-3 52 36,88	-40 24 47,96	-3,876911111	-40,41332222
16	Mulungu	Ceará	-3 56 19,3	-40 25 18,2	-3,938694444	-40,42172222
17	Varjota	Ceará	-4 4 52,2	-40 30 4,2	-4,081166667	-40,50116667
18	Cariré	Ceará	-4 1 4,93	-40 28 33,27	-4,018036111	-40,47590833
19	Moraújo	Ceará	-3 52 26,3	-40 21 58,6	-3,873972222	-40,36627778
20	Graça	Ceará	-3 42 48,12	-40 52 17,89	-3,713366667	-40,87163611
21	Hidrolândia	Ceará	-3 27 56,4	-40 41 2,8	-3,465666667	-40,68411111
22	Reriutaba	Ceará	-3 36 48,8	-40 55 52,1	-3,613555556	-40,93113889
23	Reriutaba	Ceará	-4 27 24,49	-40 43 50,38	-4,456802778	-40,73066111
24	Martinópole	Ceará	-3 43 22,5	-40 52 19,8	-3,722916667	-40,87216667
25	Reriutaba	Ceará	-4 3 43,5	-40 31 30,2	-4,062083333	-40,52505556
26	Frecheirinha	Ceará	-4 3 45,4	-40 31 23,5	-4,062611111	-40,52319444
27	Frecheirinha	Ceará	-3 43 5,4	-40 52 29,2	-3,718166667	-40,87477778
28	Groaíras	Ceará	-3 57 50,5	-40 20 22,3	-3,964027778	-40,33952778
29	Groaíras	Ceará	-3 58 37,03	-40 20 57,25	-3,976952778	-40,34923611
30	Moraújo	Ceará	-3 31 6,7	-40 36 23,3	-3,518527778	-40,60647222
31	Groaíras	Ceará	-4 1 55,94	-40 34 4,55	-4,032205556	-40,56793056
32	Cariré	Ceará	-3 41 39,3	-40 50 30,8	-3,69425	-40,84188889
33	Tianguá	Ceará	-3 59 53,8	-40 34 26,8	-3,998277778	-40,57411111
34	Moraújo	Ceará	-3 31 32,4	-40 35 48,2	-3,525666667	-40,59672222
35	Tianguá	Ceará	-4 10 10,4	-40 36 39,7	-4,169555556	-40,61102778
36	Coreaú	Ceará	-3 47 41	-40 24 9	-3,794722222	-40,4025
37	Sobral	Ceará	-3 34 15,3	-40 40 40,58	-3,570916667	-40,67793889
38	Groaíras	Ceará	-3 52 29,13	-40 21 53,08	-3,874758333	-40,36474444
39	Croatá	Ceará	-4 20 13,9	-40 51 23,9	-4,337194444	-40,85663889
40	Cariré	Ceará	-3 7 6,41	-40 50 28,9	-3,118447222	-40,84136111
41	Viçosa do Ceará	Ceará	-4 8 55,6	-41 23 37	-4,148777778	-41,39361111
42	Granja	Ceará	-3 7 8,9	-40 50 26,64	-3,119138889	-40,84073333
43	Cabeceiras do Piauí	Piauí	-3 19 18,5	-42 3 24,3	-3,321805556	-42,05675
44	Granja	Ceará	-3 50 28,11	-40 40 2,49	-3,841141667	-40,66735833
45	Croatá	Ceará	-4 22 40,87	-40 51 51,09	-4,378019444	-40,86419167
46	Murici dos Portelas	Piauí	-3 19 9,09	-42 3 29,9	-3,319191667	-42,05830556
47	Granja	Ceará	-3 9 45,14	-40 52 30,26	-3,162538889	-40,87507222
48	Lagoa do Piauí	Piauí	-5 23 6,1	-42 34 16,3	-5,385027778	-42,57119444
49	Cariré	Ceará	-5 22 29,6	-42 36 14,9	-5,374888889	-42,60413889
50	Brasileira	Piauí	-4 8 55,5	-41 23 33,7	-4,14875	-41,39269444
51	Cabeceiras do Piauí	Piauí	-3 19 9,09	-42 3 29,9	-3,319191667	-42,05830556
52	Cabeceiras do Piauí	Piauí	-3 19 12,8	-42 3 29,4	-3,320222222	-42,05816667
53	Murici dos Portelas	Piauí	-4 22 40,5	-42 26 8,6	-4,377916667	-42,43572222
54	Brasileira	Piauí	-3 33 12,6	-41 6 4,5	-3,5535	-41,10125
55	Cabeceiras do Piauí	Piauí	-4 22 58,5	-42 26 50,9	-4,382916667	-42,44747222
56	Lagoa do Piauí	Piauí	-4 0 47,66	-40 35 15,16	-4,013238889	-40,58754444
57	Murici dos Portelas	Piauí	-4 23 22,8	-42 26 34,7	-4,389666667	-42,44297222
58	Murici dos Portelas	Piauí	-4 27 40,1	-42 21 9,2	-4,461138889	-42,35255556

59	Massapê	Ceará	-3 30 13,13	-40 21 46,94	-3,503647222	-40,36303889
60	Cabeceiras do Piauí	Piauí	-4 23 33,5	-42 21 26,2	-4,392638889	-42,35727778
61	Domingos Mourão	Piauí	-4 9 14,7	-41 22 9,85	-4,154083333	-41,36940278
62	Milton Brandão	Piauí	-4 41 5,2	-41 26 55	-4,684777778	-41,44861111
63	São João do Arraial	Piauí	-3 48 43,2	-42 29 39	-3,812	-42,49416667
64	Viçosa do Ceará	Ceará	-3 34 55,86	-41 4 43,2	-3,582183333	-41,07866667
65	Coreaú	Ceará	-3 36 50,76	-40 44 28,5	-3,6141	-40,74125
66	Traipu	Alagoas	-9 55 14	-36 56 57	-9,920555556	-36,94916667
67	Traipu	Alagoas	-9 54 30	-36 57 6,7	-9,908333333	-36,95186111
68	Domingos Mourão	Piauí	-4 12 56,4	-41 26 11,7	-4,215666667	-41,43658333
